# Supplementary material for: Feedforward attentional selection in sensory cortex
Source: Nat Commun. 2023 Sep 26;14:5993. doi: 10.1038/s41467-023-41745-1 (PMC10522696; doi:10.1038/s41467-023-41745-1)
Supplement: Supplementary file 2 — Reporting Summary [file 41467_2023_41745_MOESM2_ESM.pdf]

nature portfolio

Corresponding author(s): Jacob A. Westerberg

Last updated by author(s): Jul 19, 2023

Reporting Summary

Nature Portfolio wishes to improve the reproducibility of the work that we publish. This form provides structure for consistency and transparency in reporting. For further information on Nature Portfolio policies, see our Editorial Policies and the Editorial Policy Checklist.

Statistics

For all statistical analyses, confirm that the following items are present in the figure legend, table legend, main text, or Methods section.

n/a	Confirmed	
☐	☒	The exact sample size (*n*) for each experimental group/condition, given as a discrete number and unit of measurement
☐	☒	A statement on whether measurements were taken from distinct samples or whether the same sample was measured repeatedly
☐	☒	The statistical test(s) used AND whether they are one- or two-sided<br>*Only common tests should be described solely by name; describe more complex techniques in the Methods section.*
☒	☐	A description of all covariates tested
☒	☐	A description of any assumptions or corrections, such as tests of normality and adjustment for multiple comparisons
☐	☒	A full description of the statistical parameters including central tendency (e.g. means) or other basic estimates (e.g. regression coefficient) AND variation (e.g. standard deviation) or associated estimates of uncertainty (e.g. confidence intervals)
☐	☒	For null hypothesis testing, the test statistic (e.g. *F*, *t*, *r*) with confidence intervals, effect sizes, degrees of freedom and *P* value noted<br>*Give P values as exact values whenever suitable.*
☐	☒	For Bayesian analysis, information on the choice of priors and Markov chain Monte Carlo settings
☒	☐	For hierarchical and complex designs, identification of the appropriate level for tests and full reporting of outcomes
☒	☐	Estimates of effect sizes (e.g. Cohen's *d*, Pearson's *r*), indicating how they were calculated

Our web collection on statistics for biologists contains articles on many of the points above.

Software and code

Policy information about availability of computer code

Data collection	Tucker-Davis Synapse data acquisition interface, Reflective Computing TEMPO/VideoSync behavioral control software, Eyelink eye tracking software v5.16
Data analysis	The Mathworks MATLAB R2020b, The R Foundation for Statistical Computing R v4.1.2, RStudio 2021.09.2, RStan 2.21.1, Custom code in MATLAB and R https://doi.org/10.5281/zenodo.8163978

For manuscripts utilizing custom algorithms or software that are central to the research but not yet described in published literature, software must be made available to editors and reviewers. We strongly encourage code deposition in a community repository (e.g. GitHub). See the Nature Portfolio guidelines for submitting code & software for further information.

Data

Policy information about availability of data

All manuscripts must include a data availability statement. This statement should provide the following information, where applicable:

- Accession codes, unique identifiers, or web links for publicly available datasets
- A description of any restrictions on data availability
- For clinical datasets or third party data, please ensure that the statement adheres to our policy

Data supporting the findings of the main text are openly available through Data Dryad: https://doi.org/10.5061/dryad.s1rn8pk9t.

Human research participants

Policy information about studies involving human research participants and Sex and Gender in Research.

Reporting on sex and gender	No human participants were used in this study.
Population characteristics	No human participants were used in this study.
Recruitment	No human participants were used in this study.
Ethics oversight	No human participants were used in this study.

Note that full information on the approval of the study protocol must also be provided in the manuscript.

Field-specific reporting

Please select the one below that is the best fit for your research. If you are not sure, read the appropriate sections before making your selection.

☒ Life sciences ☐ Behavioural & social sciences ☐ Ecological, evolutionary & environmental sciences

For a reference copy of the document with all sections, see nature.com/documents/nr-reporting-summary-flat.pdf

Life sciences study design

All studies must disclose on these points even when the disclosure is negative.

Sample size	Number of monkeys, sessions, and behavioral trials were chosen as to be consistent with previous studies engaging macaque monkeys in a cognitive task concomitant with laminar neurophysiological recordings. We minimize the number of animals used for ethical considerations. Two monkeys are used as that is the minimum required to observe replication of results across subjects and to justify a robust, reliable publications (Roelfsema and Treue, 2014).
Data exclusions	No data were excluded from analysis.
Replication	No previous studies have reported the documented results. Results were replicated across animals within the study where samples sizes were sufficient.
Randomization	Predefined experimental groups were not necessary for the study.
Blinding	Blinding was not relevant to the study as investigators did not need to allocate subjects to groups.

Reporting for specific materials, systems and methods

We require information from authors about some types of materials, experimental systems and methods used in many studies. Here, indicate whether each material, system or method listed is relevant to your study. If you are not sure if a list item applies to your research, read the appropriate section before selecting a response.

Materials & experimental systems

n/a	Involved in the study	
☒	☐	Antibodies
☒	☐	Eukaryotic cell lines
☒	☐	Palaeontology and archaeology
☐	☒	Animals and other organisms
☒	☐	Clinical data
☒	☐	Dual use research of concern

Methods

n/a	Involved in the study	
☒	☐	ChIP-seq
☒	☐	Flow cytometry
☒	☐	MRI-based neuroimaging

Animals and other research organisms

Policy information about studies involving animals; ARRIVE guidelines recommended for reporting animal research, and Sex and Gender in Research

Laboratory animals	Two male macaque monkeys (Macaca radiata; monkey Ca [age: 14 years], monkey He [age: 12 years]) were used in this study.
Wild animals	No wild animals were used in this study.
Reporting on sex	Sex-based analyses were not performed in this study as the only available subjects were male macaques.
Field-collected samples	No field-collected samples were used in this study.
Ethics oversight	All procedures were in accordance with the National Institutes of Health Guidelines and the Association for Assessment and Accreditation of Laboratory Animal Care International's Guide for the Care and Use of Laboratory Animals and approved by the Vanderbilt Institutional Animal Care and Use Committee in accordance with United States Department of Agriculture and United States Public Health Service policies.

Note that full information on the approval of the study protocol must also be provided in the manuscript.
